# MIL-53 MOF on Sustainable Biomaterial for Antimicrobial Evaluation Against *E. coli* and *S. aureus* Bacteria by Efficient Release of Penicillin G

**DOI:** 10.3390/jfb16080295

**Published:** 2025-08-15

**Authors:** Delia Monserrat Ávila-Márquez, Alien Blanco Flores, Helen Paola Toledo Jaldin, Mateo Burke Irazoque, Maribel González Torres, Alfredo Rafael Vilchis-Nestor, Carla Calderon Toledo, Sergio Gutiérrez-Cortez, Juan Pablo Díaz Rodríguez, Alejandro Dorazco-González

**Affiliations:** 1 Mechanical Engineering Division, Technological of Superior Studies of Tianguistenco, National Technological of Mexico, Santiago Tianguistenco 52650, Mexico; deliam_am@test.edu.mx (D.M.Á.-M.); helen_mecanica@test.edu.mx (H.P.T.J.); maribel.gonzalez@test.edu.mx (M.G.T.); juan_202021055@test.edu.mx (J.P.D.R.); 2 Institute of Metallurgy, Universidad Autónoma de San Luis Potosí, San Luis Potosí 78210, Mexico; 3 Environmental Microbiology Unit, Institute of Molecular Biology and Biotechnology (IBMB), Universidad Mayor de San Andrés, La Paz 0201-0220, Bolivia; mburkei2@fcpn.edu.bo (M.B.I.); cvcalderon@fcpn.edu.bo (C.C.T.); sgutierrezc@fcpn.edu.bo (S.G.-C.); 4 Joint Center for Research in Sustainable Chemistry UAEM-UNAM, (CCIQS), Toluca 50200, Mexico; arvilchisn@uaemex.mx; 5 Institute of Chemistry, National Autonomous University of Mexico, Mexico City 04510, Mexico

**Keywords:** MIL series synthesis, advanced materials characterization, MIL-53, biphasic calcium phosphate, penicillin G loading and release

## Abstract

The development of efficient antibiotic-releasing materials derived from sustainable and recyclable compounds represents a key area within biomedical materials science, particularly in the treatment of antibacterial infections. Herein, a Fe^3+^/terephthalate-based metal–organic framework (**MIL-53**) and a novel advanced material made of **MIL-53** with biogenic hydroxyapatite (**1**) were prepared by solvothermal reactions, and these were studied in detail as a Penicillin-G-releasing material. After loading Penicillin G on **1** and **MIL-53**, the antibiotic percentage release was studied, and the antimicrobial effectiveness of each material was evaluated against two bacterial ATCC strains (*E. coli* and *S. aureus*) and various Penicillin-G-resistant uropathogenic strains such as *E. coli* isolates (HHM 25, ERV 6, and FGI 4). Functional, structural, and morphological characteristics of these materials were thoroughly studied by analytical tools (FTIR, XRD, BET, SEM-EDS, and XPS). The Penicillin G load did not exceed 50% in both materials. The Penicillin G adsorption mechanism involves several types of interactions with the materials. The release of the antibiotic was more efficient from **MIL-53**, where the load did not exceed 20%. The release was analyzed using mathematical models. They indicated that when Penicillin G is released from **MIL-53**, the process follows diffusion through a uniform matrix; however, **1** is more porous, which helps with the release by diffusion of Penicillin G, and **1** exhibits more than a 90% inhibition of the growth of bacteria and strains like **MIL-53**. This suggests a valuable approach to antibiotic activity against resistant pathogens. The use of composite materials derived from the Fe-MOF with a sustainable matrix of hydroxyapatite as antibiotic-releasing materials has been unexplored until now.

## 1. Introduction

Although antibiotics have saved millions of lives from infections, their excessive and inadequate use has increased over the past decade. Often, the sick person decides to stop treatment before finishing the full cycle, increasing the risk of the bacteria becoming resistant to future medications. In 2019, this situation caused 1.27 million deaths in the world. This number is expected to rise to 10 million per year by 2050, which would far exceed cancer-related deaths [1]. In 2023, the Institute for Health Metrics and Evaluation (IHME) reported that two out of five deaths in the Americas were associated with antimicrobial resistance in 2019; these data correspond to 11.5% of deaths worldwide. More conclusive data indicate that 569,000 deaths were related to bacterial antimicrobial resistance in the 35 countries of the Americas Region, according to the World Health Organization (WHO) [2]. The WHO has declared that antimicrobial resistance is one of the top 10 global health problems and must be addressed urgently [3]. Antibiotic resistance is a global health threat because it causes problems such as pneumonia, urinary tract infections, or surgical wounds, which are becoming difficult or impossible to treat. The complications arising from these problems are that the treatments prescribed by doctors are increasingly prolonged, costly, and toxic. In addition to the increasing number of hospitalizations, postoperative complications, nosocomial infections, etc., are on the rise [4].

The situation is serious because antimicrobial resistance becomes worse while new drugs are not being developed quickly enough to combat the most dangerous and deadly bacteria. New alternatives must be proposed to address this problem. The solution may lie in the Materials Science and Engineering field. One strategy could be to combine the antibiotic with an advanced material with specific characteristics and properties, allowing the controlled release of antibiotics within the bacteria, simulating the “Trojan horse” effect [5].

For this purpose, metal–organic frameworks (MOFs) can be employed. MOFs are a potential alternative for drug delivery because they can be synthesized with the required characteristics, such as structural compatibility (adequate pore size to adsorb the drug in the pores), chemical stability (in water and physiological media), ability to interact with the drug (through functional groups or the metal center), biocompatibility (ligand and non-toxic metal center), high loading capacity, and possibility of sustained release [6].

In this regard, Gautam et al. [7] report that MOFs have an efficient drug loading capacity. They can be loaded with efficiencies close to 100%, unlike other materials that have low loading percentages. For example, non-functionalized mesoporous silica (SBA-15) has a low release percentage between 1 and 4% [8], and non-functionalized ZIF 8 (5 FU), although it showed loading values of 12%, still has a low value [9]. Another example of these materials is the hydroxyapatite–gelatin (HAp-GEL) polymer composite, the loading of which was estimated to be approximately 5.9% [10].

The most relevant MOFs for drug release are related to the assembly of Fe^3+^ metal clusters, according to Rojas et al. [11]. The chemical structure of MOFs allows blood compatibility, while their functional group has a high polarity. They can be easily eliminated through urine and feces [12,13].

One of the most common types of MOFs is the MIL (Materials of Institute Lavoisier Frameworks) series. These possess a different porosity and a large specific surface area, with abundant active sites that promote a high adsorption capacity. They are stable under various conditions and media, heat, air, water, or robust solutions, among others. One of the most relevant characteristics is the multidimensional network structure that allows the adsorption of diverse organic molecules. They also have functional groups classified as unsaturated Lewis acids used in adsorption processes [14]. Examples of MOFs belonging to this group are **MIL-53**. To obtain this MOF, organic (terephthalic acid) and Fe^3+^ ions are combined, generating a hybrid material whose organic unit provides flexibility and functionality [15]. The Fe^3+^ provides high structural, mechanical, and thermal stability, making it practical for drug liberation. The synthesis of **MIL-53** is reproducible and scalable at the industrial level, and the solvent is not toxic, which is preferred for medical applications [16]. In the case of using any organic solvent with a certain degree of toxicity, this is eliminated from the medium once the MOFs are obtained, through successive washes and even an activation process at different temperatures [17].

Another alternative that is often used, together with successive washes to remove the solvent, is to grow the MOF on a matrix (composite formation). In this case, the proposal brings benefits such as retention of a smaller amount of solvent, since the solvent is “diluted” because the MOF is dispersed on the surface of the matrix. Sometimes, as when it is usually supported on hydroxyapatite, a higher thermal and chemical stability is incorporated, since even if the thermal treatment becomes more aggressive, it does not usually collapse the MOF structure, and this also favors the elimination of trapped solvents. Additional advantages are that the overall biocompatibility of the system could be improved, especially when antibiotics are released. Hydroxyapatite is a good matrix candidate for these applications [18].

Composite materials are essential because they generate synergic effects, making them more competitive in the medical and biotechnology fields. Biodegradable materials have additional benefits and are an alternative to designed drug release systems. One source of raw material for biomaterials is solid waste [19]. In addition, waste is considered a very valuable resource for the circular economy and the environment’s sustainability [20]. Hydroxyapatite (HAp) is a bioactive, biocompatible, and biodegradable material obtained from natural sources like animal bones. It is derived from waste of the food industry and household activities. Globally, it is reported that the generation of this type of waste reaches 30 million metric tons worldwide. But this is not the only biomaterial that can be obtained; β-tricalcium phosphate (β-Ca_3_(PO_4_)_2_) is another inorganic compound obtained from bones when they are subjected to heat treatment at high temperatures (above 100 degrees Celsius). HAp and β-tricalcium phosphate form a material named biphasic calcium phosphate, which is used as a biomaterial in biomedical applications, specifically in cements for orthopedic and dentistry, according to Moreno et al. [21]. However, it has not been used for other biomedical applications such as drug release.

Another biomaterial that has been reported upon is biogenic hydroxyapatite (**BCaP**); although it is showing a trend in its use for medical applications such as coating and support in bone regeneration [22], no specific studies have been found where the it has been used for loading and release of Penicillin G (only subjected to washing with hot water and a mixture of organic solvents and without calcination), and it is much less supported in materials such as MOFs. **BCaP** is obtained by a low-cost method from an economic and energetic point of view, since it does not require calcination stages or high temperatures. Also, it is known that the methods to obtain HAp are only methods that involve high energy consumption [22,23,24].

When enterobacteria require a source of Fe^3+^, they release an organic compound named enterobactin; therefore, the organism can trap it. Thus, this compound crosses the cell wall of the bacteria and releases iron ions. Once it binds to Fe^3+^, the organic compound forms a MOF-like material [25]. It is therefore deduced that this could be the principle of releasing antibiotics that are no longer as efficient alone, but combined with these materials, could lead to the death of microorganisms such as *E. Coli* and *S. Aureus* [26], among others. Therefore, it is essential to study both parts (metal ions and organic ligands) in the release of simple and well-studied antibiotic molecules such as penicillin.

This work describes the results obtained by focusing on the analysis of Fe^3+^ when it forms a metal–organic network (**MIL-53**) for the controlled release of Penicillin G, as well as the evaluation of the spectrum of antibacterial efficacy against bacterial strains sensitive and resistant to the antibiotics. Additionally, it was analyzed whether the presence of **MIL-53** deposited in a **BCaP** could be more efficient for antibiotic release.

**MIL-53** has been previously reported for different applications; however, there is no evidence that it has grown on a sustainable biomaterial based on biogenic HAp. It is important to highlight that this is the first time that a material like **1** has been studied for its antibiotic adsorption capacity, release, and antibacterial activity against several strains resistant to penicillin.

## 2. Materials and Methods

### 2.1. Materials

All reagents used in this research were analytical grade. N,N-dimethylformamide (DMF), terephthalic acid (H_2_BDC), and Penicillin G (96–100% purity) were purchased from Sigma-Aldrich. FeCl_3_·6H_2_O (98% of purity) and methanol were purchased from Wholer, and a Regulatory solution (PBS) was purchased from J.T Baker. All experiments were carried out with deionized water. All glassware is rinsed with royal water and deionized water before use. The filters used were syringe filters with sterile 0.2 µm SFCA/PF membrane. All the reagents and materials were purchased by branches of the companies I mention. In this case the city is Mexico City, Mexico.

### 2.2. Procedure to Obtain ***BCaP*** Biomaterial from Beef Bone Powder, Synthesis of ***MIL-53***, and ***1***

**Figure 1:**
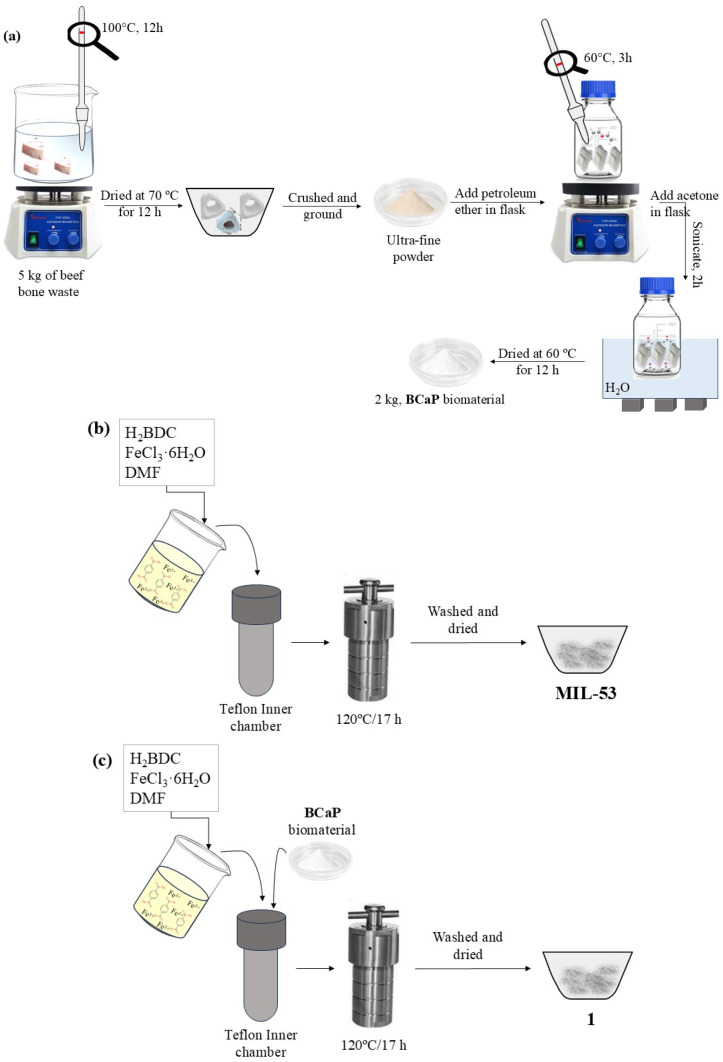
(**a**) **BCaP** biomaterial preparation, (**b**) synthesis of **MIL-53**, and (**c**) **1** hybrid material.

The beef bone (from Toluca Municipal Slaughterhouse, Toluca, Mexico) was collected and boiled in water until the visual presence of organic matter (fat) was eliminated. The process was repeated until the water was clear. Subsequently, the bone was dried at 70 °C for 12 h. After this time, it was crushed and ground into an ultra-fine powder. A total of 5.0 g of powder was placed in paper filter bags and inserted into a flask with petroleum ether. The system was heated to 60 °C for 3 h. The bottle was uncovered every 30 min to expel the generated gases. Subsequently, the bags were removed, opened, and dried at room temperature. The procedure was repeated twice. The third stage of the treatment consisted of reproducing the same procedure mentioned above, but with acetone instead of petroleum ether, and sonicated for two hours. Every 15 min, the bottle was opened to release gases. After this time, the bags were removed, opened, and dried at 60 °C for 12 h (Figure 1a). The procedure was repeated twice. Finally, the powder obtained was labeled **BCaP**. In the process, for every 5 kg of bone, 3 kg of **BCaP** powder was obtained (Figure 1a), representing a 60% yield. The loss (40%) was mainly due to the crushing and pulverization process of the biomaterial.

**MIL-53** was synthesized by a solvothermal method according to Jakhar et al. [27]. In a Teflon inner chamber, the solvent, precursor of the metal center, and the organic ligand were put in contact with each other for a certain time at 120 °C. After this time, the heating was stopped, and the autoclave was allowed to reach room temperature. The mixture was then gravity filtered, washed to remove solvent residues, and dried for further use (Figure 1b).

To grow **MIL-53** on **BCaP**, the same **MIL-53** synthesis procedure was followed, but an additional 50 mg of BCaP was added to the mixture. The material resulting from the synthesis will hereinafter be referred to as **1** (Figure 1c).

### 2.3. Characterization of ***BCaP***, ***MIL-53***, and ***1***

The surface morphology of materials was analyzed by Scanning Electron Microscopy (SEM) using a JEOL JSM-610LV microscope (JEOL, Tokyo, Japan) operated at 20 or 15 kV and equipped with a Bruker QUANTAX 200 Energy-Dispersive X-ray Spectrometer (EDS) (Bruker, Billerica, MA, USA) for elemental characterization. All samples were fixed on a support with a carbon film and sputter-coated with platinum. Fourier Transform Infrared (FTIR) absorption spectra identified functional groups of synthesized materials, which were recorded in the 4000–500 cm^−1^ range in a Bruker Tensor 27 FT-IR spectrophotometer. The species formed were identified by powder X-ray diffraction (XRD) in a Bruker D8 Advance X-ray diffractometer equipped with a CuK radiation source and SOL-X solid-state detector. Analyses were performed in a 2θ range from 10° to 90°, with a counting time of 0.3 s per step. This equipment was purchased by the Joint Research Center for Sustainable Chemistry (CCIQS), UAEM/UNAM, Toluca, Mexico. Textural properties were obtained by the BET technique of N_2_ physisorption at 77 K. Before data adsorption collection, 126.7 and 241.8 mg of the MIL-53 and 1 sample, respectively, were degassed at 180 °C for 24 h. The specific surface area (S_e_) was determined by the equation of Brunnauer–Emmet–Teller (BET). The adsorption–desorption isotherms were obtained by plotting the adsorbed volume of nitrogen under standard conditions of temperature and pressure versus the relative pressure P/P0 to determine the pore size and estimate the shape of the pores according to the International Union of Pure Applied Chemistry (IUPAC) rules [28]. X-ray photoelectron spectroscopy (XPS) was performed on an X Thermo Scientific model K-Alpha (Waltham, MA, USA) using Al Ka radiation (1486 eV) generated at 120 W. All binding energies were calibrated for the C1s peak (285 eV) arising from adventitious carbon. The spectra were analyzed using AVANTAGE, v4.87 software. The background subtraction was performed using the Shirley method; the GaussLorentz method was used whenever curve fitting was needed.

Additionally, samples with Penicillin G loaded were characterized by SEM, XRD, and XPS techniques.

### 2.4. Penicillin G Loading in ***BCaP***, ***MIL-53***, and ***1***

Penicillin G loading was achieved by the incipient wetness impregnation procedure reported by Gordon et al. [29]. A total of 1 mL of 20 mg/mL Penicillin G solution was added to 100 mg of the chosen material powder, and then the mixture was mixed until homogenized. Then, it was placed in a vacuum at 70 °C for 24 h to remove the excess solvent. The loading amount of Penicillin G as a percentage (P_load_ (%)) was calculated by Equation (1).

(1) $$P_{\mathrm{load}}(\%)=\frac{W_{\mathrm{Penicillin}\;G}}{W_{\mathrm{materials}}}\times 100,$$

where W_Penicillin G_ is the weight of Penicillin G in the materials synthesized, and W_materials_ is the weight of materials synthesized.

The antibiotic loading experiments on the three materials were performed in triplicate. The standard deviation (s) was calculated through Equation (2):

(2) $$s=\frac{1}{n-1}\sum_{i=1}^{n}{\left(x_{i}-\bar{x}\right)}^{2},$$

where x_i_ is the individual load value, $\bar{x}$ is the average load value, and n is the number of repetitions (n = 3).

The materials with loaded Penicillin G (**Pen**) were named **BCaP-Pen**, **MIL-53-Pen**, and **1-Pen**. The former is the MOF with the loaded Penicillin G, and the latter is the composite with the loaded Penicillin G.

The release studies were carried out in the same way for each material. A total of 5.0 mg of materials loaded with Penicillin G were suspended in 15 mL PBS solution at 37 °C. Then, the mixture was stirred at 120 rpm. At each time interval, 2 mL of the mixture was taken with a syringe, and the samples were filtered with a 0.45 µm PTFE membrane. The same volume was replaced in the sample with 2 mL of fresh PBS. Penicillin G was quantified using a Velab brand spectrophotometer, obtaining the absorption spectrum for each sample at different time intervals.

A standard calibration curve was obtained and the Adj. R-square (R^2^) was 0.9980, which is a value that indicates an excellent linearity of the obtained straight lines. The absorbance values were obtained for a maximum wavelength (λ_max_) of 225 nm. The linearity range was between 1 and 30 mg/L of the initial concentration of Penicillin G.

The released percentage (%) of Penicillin G (P_release_) was calculated according to Equation (3):

(3) $$P_{\mathrm{release}}(\%)=\left(\frac{M_{t}}{M_{0}}\right)\times 100,$$

where the amount of Penicillin G released at different time intervals is M_t_ (mg). Additionally, the amount of Penicillin G loaded within materials synthesized (M_0_) in mg was determined by the mass difference method, where the amount was calculated by the difference between the mass of the material before loading the drug and the mass of the material after loading the drug [30].

### 2.5. Bacterial Strains

Bacterial strains for this study comprised both Penicillin-G-resistant and sensitive strains and were used to assess the spectrum of antibacterial efficacy of **MIL-53-Pen** and **1-Pen**. Three clinical isolates of UPEC (Uropathogenic Escherichia coli), HHM 25, ERV 6, and FGI 4, were chosen for their demonstrated resistance to Penicillin G. Additionally, two Penicillin-G-sensitive strains were selected from the American Type Culture Collection: S. aureus ATCC 29213 and E. coli ATCC 25922.

### 2.6. Antibacterial Activity Test

Antibacterial activity of **MIL-53-Pen** and **1-Pen** was assessed by counting the Colony Forming Units per mL, modified from Hatamie et al. [31]. Briefly, isolated bacterial colonies were grown in LB broth for 2–3 h. Following incubation, the optical density (OD) at 600 nm was measured, and the concentrations were adjusted to 0.5 McFarland (108 cell/mL). In sterile conditions, 100 µL of the adjusted cultures were mixed with 900 µL of LB broth containing MOFs at a concentration of 20 µg/mL [32]. Controls included a growth control, which contained the bacterial cultures in LB broth without MOFs, and a Penicillin G control, which contained bacterial cultures in LB broth without MOFs but supplemented with Penicillin G at concentrations equivalent to those present in the MOF formulations. The mixtures were incubated with agitation at 37 °C and 320 rpm for an 18 h period. Following incubation, serial dilutions were performed up to 10^−6^ for the samples with the nanomaterials and antibiotic, and up to 10^−7^ for the growth control. A volume of 100 µL from each dilution was then plated on LB agar plates and spread evenly using sterilized beads. After discarding the beads, the plates were incubated at 37 °C for 18 h, allowing for colony growth and subsequent enumeration. Each treatment was evaluated in triplicate; furthermore, the whole experimental and control setup was replicated three times to confirm the reproducibility of the results.

The statistical significance of differences observed between treated samples and penicillin controls was determined through the Student’s *t*-test, with a *p*-value of less than 0.05 considered indicative of significant antibacterial activity.

## 3. Results and Discussion

### 3.1. Materials Characterization

**Figure 2:**
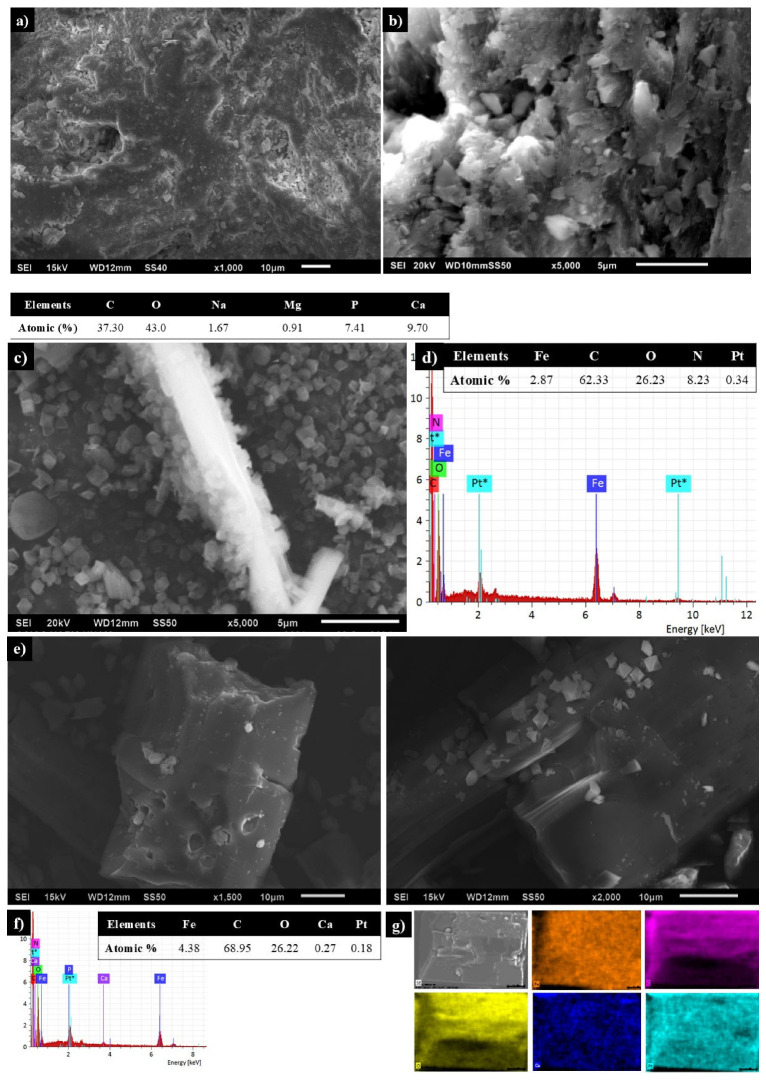
SEM micrographs of (**a**,**b**) **BCaP** biomaterial, (**c**) **MIL-53**, (**d**) EDS of **MIL-53,** (**e**) **1**, (**f**) EDS, and (**g**) chemical mapping of **1** (Fe: orange, C: violet, O: yellow, Ca: dark blue and Pt*: light blue, the latter is the material that was used to coat the samples in the SEM analysis).

Micrographs of **BCaP** show a rough surface with cavities that can be considered large pores (Figure 2a), and the particles have a plate morphology (Figure 2b). EDS analysis confirms the presence of element characteristics of this material: P and Ca, and to a lesser extent other metal ions (Na^+^ and Mg^2+^) [33].

**MIL-53** particles have a smooth surface and are irregular in size, with an octahedron-type structure (Figure 2c). Chemical composition revealed the presence of metallic elements (Fe) and non-metallics (C, O), and the latter is characteristic of the organic ligand (Figure 2d).

SEM images of **1** showed smaller and larger **MIL-53** particles when they grew on **BCaP**, although the smooth surface and octahedron-type structure were the same (Figure 2e). EDS confirms the formation of **1** (Figure 2f). Chemical mapping showed a homogeneous distribution of **MIL-53** over the surface of the **BCaP** (Figure 2g).

**Figure 3:**
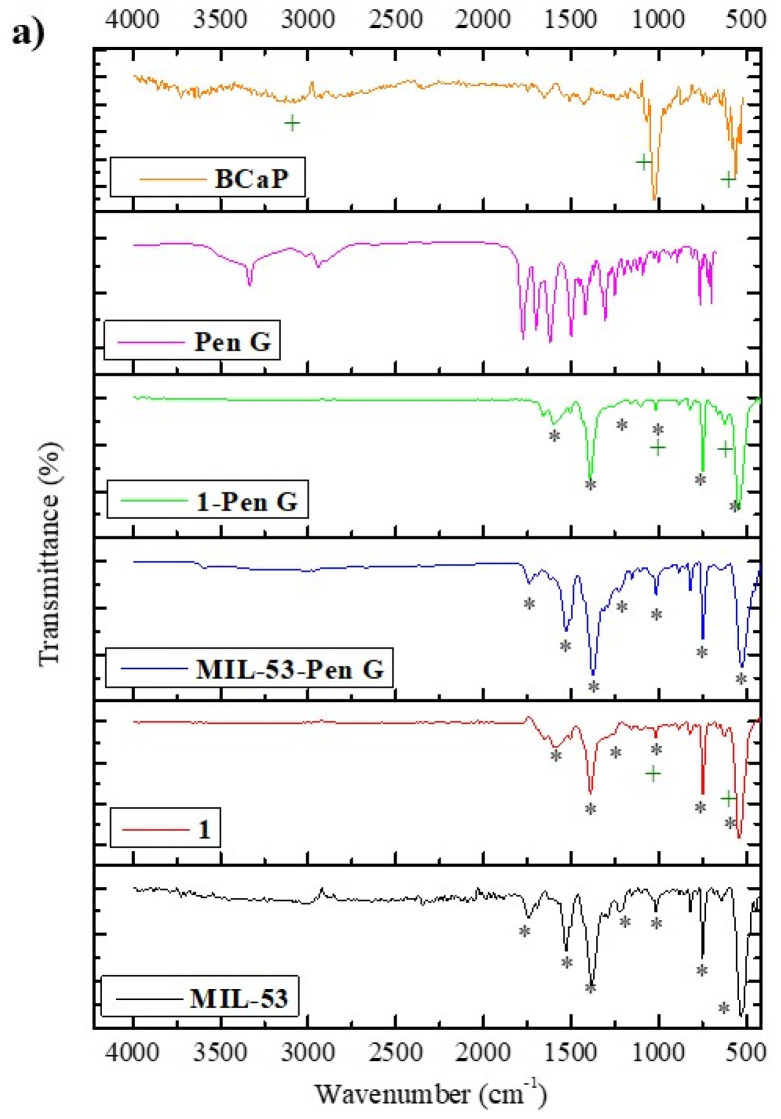

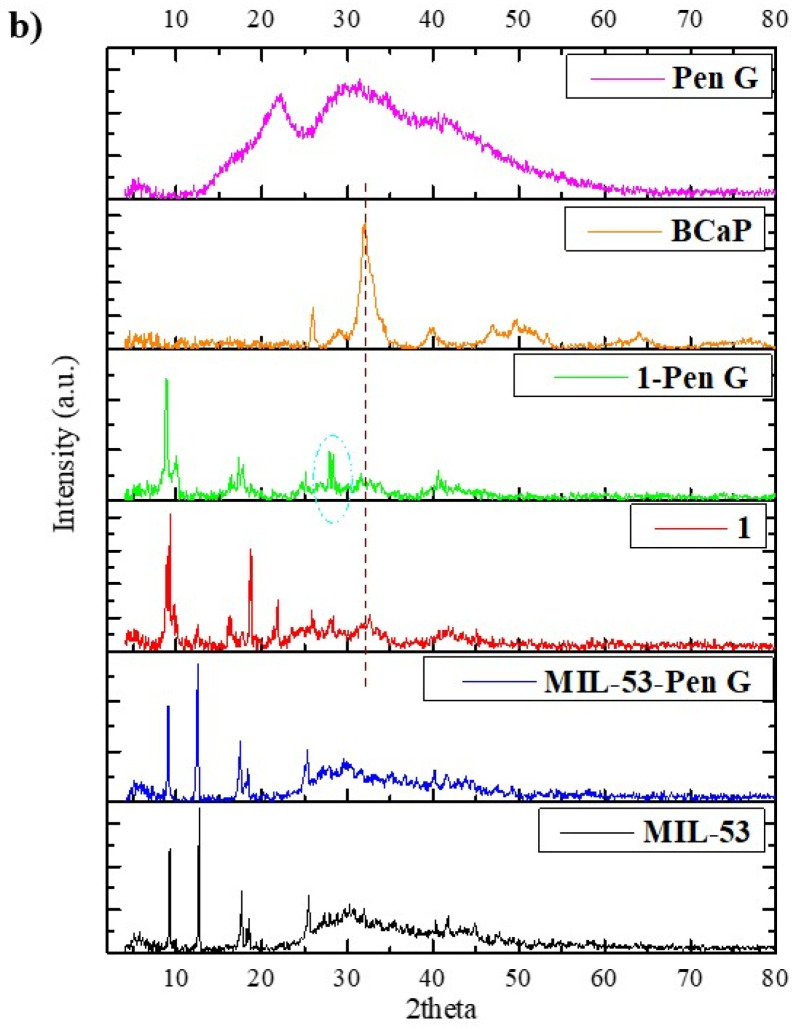
(**a**) FTIR spectra and (**b**) XRD of **BCaP**, **1**, and **MIL-53** materials, and these materials with **Pen** loaded. The symbols in the Figure represent common or coinciding peaks in each spectrum.

In Figure 3a, the spectra of the materials show functional groups corresponding to **BCaP**, **1**, and **MIL-53**. **BCaP** shows a signal at 570 cm^−1^ and other peaks between 600 and 500 cm^−1^, which are attributed to the symmetrical bending of PO_4_^3−^ groups, corroborating the presence of hydroxyapatite free of organic matter [34]. At 1030.50 cm^−1^, another characteristic signal (asymmetric stretching vibrations) of PO_4_^3−^ groups in HAp appears [35]. The weak band at 1433.55 cm^−1^ is correlated with CO_3_^2−^. Also, a weak intense band at 1659 cm^−1^ is associated with the -OH group of HAp [36,37]. According to the results, **BCaP** is biogenic or carbonate hydroxyapatite (HAp: Ca_10_(PO_4_)_6_(OH)_2_). In this one, the presence of CO_3_^2−^ partially substitutes the -OH or PO_4_^3−^, and last one is the most common substitution in beef bone, which has not been submitted to a thermal treatment, nor to the reaction with strong acids. This is also reported by Bano et al. [38].

FTIR of **MIL-53** showed defined and intense bands, probably due to the experimental MOF synthesized conditions (such as the solvent) [39]. A band was observed at 530.27 cm^−1^ and another was observed at 626.08 cm^−1^; both signals were attributed to the metallic cluster. The first signal was attributed to the stretching vibration of the Fe–O bond. At the same time, the second is assigned to form a Fe-oxo cluster or ring between the trivalent metal ion (Fe^3+^) and the carboxyl group of the organic ligand (H_2_BDC) [40]. Peaks between 1300 and 1670 cm^−1^ are characteristics of a dicarboxylate linker (−COO), according to Van Tran et al., [41]. Another signal at 733.23 cm^−1^ is assigned to C-H of the aromatic ring. A small and weak band at 1750 cm^−1^ is associated with a free H_2_BDC ligand [42].

The spectrum of **1** shows characteristic bands identified in the **BCaP** and **MIL-53** materials, indicating that this material is a combination of the other two. Although the peaks at 1216 cm^−1^ in **MIL-53** widen and lose intensity when **1** is formed, for the signals at 1522.90 cm^−1^ and 1747.11 cm^−1^, the same behavior is observed, allowing us to infer that there was interaction between **MIL-53** and **BCaP**, such as, for example, the anchoring of Fe ions in PO_4_^3−^ sites of **BCaP**.

The XRD pattern of **BCaP** shows a low crystallinity, since the peaks are broad (Figure 3b). There are three prominent peaks at 2θ values: 25.92°, 31.95°, and 39.92°, corresponding to HAp with crystalline planes (002), (211), and (310) [43]; it could be inferred from this that HAp should be the majority phase in **BCaP**. The presence of other much less intense peaks may be due to the presence of crystalline impurities characteristic of this type of HAp, such as variable carbonation, presence of other substituent ions, etc. [44].

In Figure 3b, the powder X-ray diffractogram of **MIL-53** shows peaks at low angle values (9.31°, 12.74°, 17.60°, 18.55°, 25.54°), which are characteristic of this MOF, according to what has been reported in the literature [45]. These peaks correspond to the orthorhombic phase.

In the diffractogram of **1**, the main peaks of **BCaP** and **MIL-53** appear at the same position, although two new signals are identified at 16.24° and 21.61° (Figure 3b). This suggests a possible structural variation of **1** concerning the MOF. The functional groups and the metal center of the MOF could probably react or combine with the main characteristic groups of the **BCaP**, leading to the formation of a non-crystalline amorphous or hybrid phase, such as the interaction of Fe^3+^ with the phosphate ions of BCaP. Other authors have claimed that it should be due to several aspects: there may be decreased crystallinity of the MOF, or it may have incomplete growth on the rough surface of BCaP, which is logical, as it could happen due to the presence of carbonate ions present in BCaP that may interfere with the self-organization of the MOF [46].

Surface-specific areas of **BCaP**, **MIL-53**, and **1** were 2.73, 38.3, and 20.2 m^2^/g, respectively. All these values are considered very low for this variable (S_e_). The average pore size was 10.43 nm and 13.11 nm for **MIL-53** and **1,** respectively, consistent with mesoporous materials. **BCaP** textural properties are improved because the porosity increased for **MIL-53** and **1**, although the pore size for the last one was higher than for **MIL-53**. According to the calculated Penicillin G molecule dimension (11.35 Å), **1** is probably the one that should support the greatest load of this antibiotic, but this would have to be confirmed with penicillin loading results; however, the release might not be entirely favored in this material. This could affect the release of the drug, considering that the pore size became larger.

### 3.2. Penicillin G Loading and Release in the Synthesized Materials

**Figure 4:**
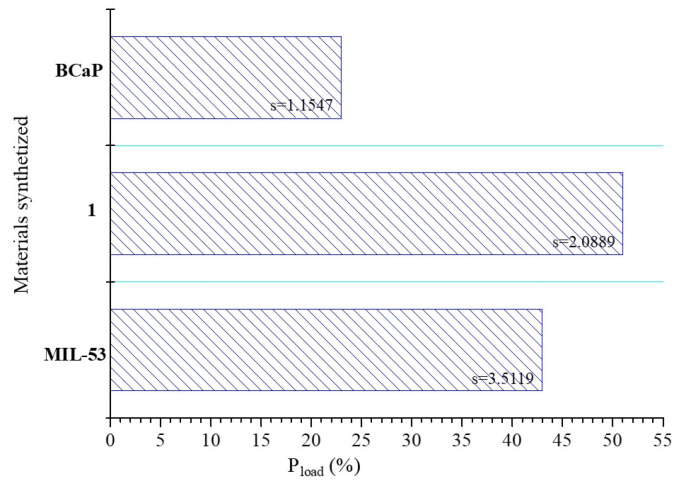
Penicillin G loaded (%) into the synthesized materials.

The loading percentage of Penicillin G on materials varied in the following order: **1** (57%) > **MIL-53** (35%) > **BCaP** (23%) (Figure 4). It is expected that **MIL-53** achieves a higher loading percentage than **BCaP**, considering that its structure is more porous (Figure 2) and that the textural properties are different. This could promote the adsorption of the antibiotic. For all materials, these percentages are higher than those reported in other works (less than 5%) [6].

For example, Ho et al. [47] reported a Penicillin G loading capacity of 0.5 mg/mg using carboxycellulose acetate nanoparticles.

Other authors have evaluated MOFs for the transport of anti-cancer drugs with satisfactory results, not only in the loading process but also in the release profiles [48,49,50].

When **MIL-53** and **1** are loaded with Penicillin G, the bands between 700 and 1800 cm^−1^ change in intensity, and there is a slight shift (Figure 3a), which means that the penicillin was deposited, considering also that the bands match the characteristic bands of Penicillin G [51]. The XRD analysis (Figure 3b) confirms the same. The diffraction peaks decrease, while their intensity and position suffer a slight shift. In the case of **MIL-53**, even the main peak disappears. This effect is more remarkable when the MOF is deposited on the **BCaP**. When Penicillin G is loaded in **1**, the peak at 31.95° disappears and another appears at 28.17° (Figure 3b). The disappearance of peaks may be due to the antibiotic being in an amorphous or disordered phase after encapsulation, and the decrease in peak intensity could be because the pores became saturated with pores. If Penicillin G interacted with the MOF, for example, new hybrid phase formation could have taken place, and the slight broadening of the **MIL-53** peaks was due to the PenG loading reducing the crystallinity of the MOF. All points have been reported by Sutherland, Moaty et al., and Huang et al. [52,53,54].

**Figure 5:**
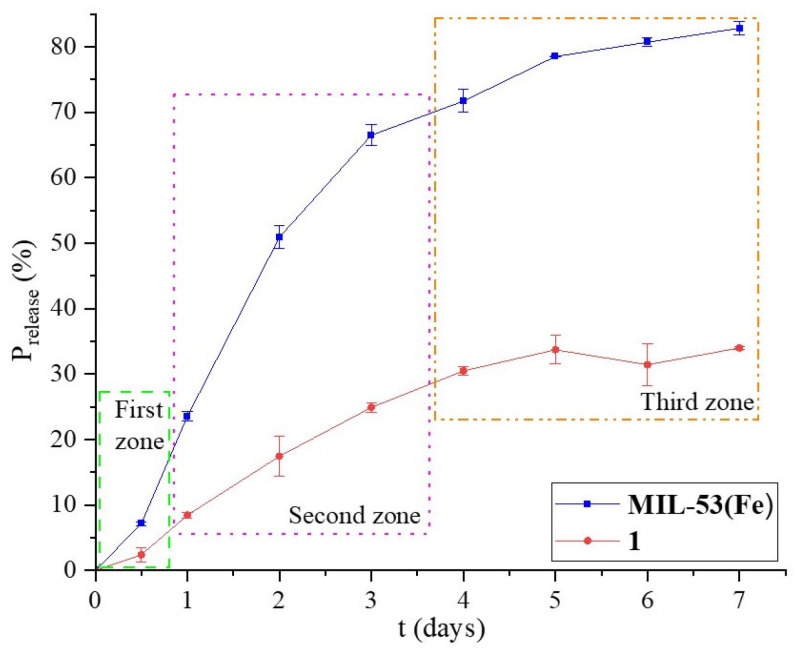
Percentage of Penicillin G released in **MIL-53** and **1** materials for 7 days in a PBS at pH 7.4.

An in vitro release study was performed using a PBS solution to simulate the body fluid, which allowed us to obtain the release kinetics. In this sense, Figure 5 shows the profile of Penicillin G release percent (%R) versus time (t). On the first day, the first zone appears with a slight inflection point. A second zone can be identified until day 3, when the release is slower and the change in the slope of the line is observed. Finally, the third zone is where the release is much slower, but it still happens. This behavior must be related to the antibiotic diffusion through different porosities in the material.

The release rate of **1** is slower than that of **MIL-53** in the first and second zones, where the slope of these curves decreases, although the %R is lower for the first material. The pore size is an important variable in the drug release process; the presence of small mesopores makes the release neither as fast nor as slow. When **MIL-53** grew on **BCaP**, the textural and morphological properties changed slightly, and **MIL-53** released 2.47 times more Penicillin G than **1**, for pore sizes of 10.43 nm and 13.11 nm, respectively.

The burst effect is an important variable to consider for drug release. If that happens, the patient may be intoxicated or poisoned by a local or temporary overdose of the medication. From an economic point of view, this effect is disadvantageous due to the expense involved in the drug. When the drug is released into the body, it is metabolized without a therapeutic effect.

The shape of the release graphs obtained indicates that a burst release does not occur in any of the four cases, according to Huang et al. [55]. This is probably because the porosity is less than what is required.

To analyze whether this effect occurs, the degree of burst (DB) was calculated, according to Brazel et al. [56], by Equation (4):

(4) $$DB=\frac{{\left(\frac{∆M_{t}}{∆t}\right)}_{t<equilibrium\;time}}{{\left(\frac{∆M_{t}}{∆t}\right)}_{SS}},$$

where the numerator refers to the initial release rate of penicillin and the denominator refers to the sustained release rate after the burst effect has decreased and the rate reaches a steady state.

Table 1 shows the values of DB; these values show that 28% of the antibiotic was released on the first day after the start of the release study for **MIL-53** and **1**. Therefore, it might work better to support the drug carrier in a matrix whose porosity helps make the release more controlled and sustained.

Different mathematical models can be used to better understand the Penicillin-G-release phenomenon. It is possible to predict the release mechanisms and the concentration of the drug in the body over time, which determines the biological efficacy of the developed DDS. The conventional models used in this study were zero-order, first-order, and Higuchi models [57]. The results of applying these models are shown in Table 1. The First-Order model was found to fit the experimental data of **MIL-53** and **1** well. This means the Penicillin-G-release process occurs through a porous matrix that does not swell. Analyzing the best fit with the application of the material, these results are correctly coherent according to the type of medication that is desired to be released. In general, the use of beta-lactam antibiotics, such as Penicillin G, requires rapid release to control serious or acute infections, to control the infection and avoid complications. With this proposed release material, intravenous injections would be avoided, which are the alternative when the antibiotic is desired to be released quickly. It is known that the injection option is not the most accepted by patients.

### 3.3. Penicillin G Loading Mechanism in the Synthesized Materials

**Figure 6:**
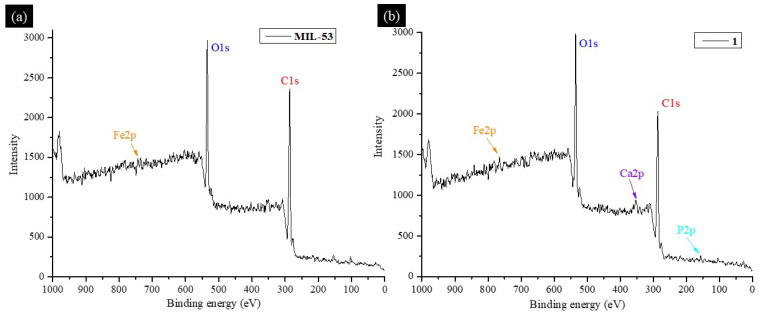

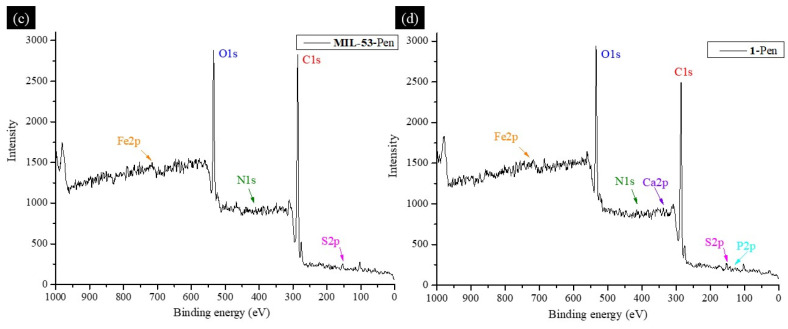
XPS survey spectrum of (**a**) **MIL-53**, (**b**) **1**, (**c**) **MIL-53-Pen**, and (**d**) **1-Pen**.

XPS spectra revealed peaks associated with Fe, C, and O for **MIL-53** (Figure 6a) and Fe, C, O, Ca, and P for **1** (Figure 6b), indicating the synthesis of this MOF and its deposition onto the BCaP biomaterial. When Penicillin G (**Pen**) was loaded on the material, the XPS survey showed signals characteristic of N and S [58], which are part of the chemical elements of the Penicillin G molecule (C_16_H_18_N_2_O_4_S).

All peaks coincide with what has been reported in the literature. By comparing XPS spectra of C 1s for **MIL-53** and **MIL-53-Pen**, they can be deconvoluted into several peaks related to bonds C-C, C-H, C-S, C-N, C=O, C-O, and a phenyl group [59,60,61]. The difference between the two spectra is in the slight shift of the signals concerning the material without Penicillin G loading and the appearance of peaks corresponding to C-N and C-S bonds. A new peak suggests (C-S) that the penicillin was indeed loaded. The peaks coincide with what has been reported in the literature [62].

Signals related to C1s, O1s, Fe2p, N1s, P2p, and Ca2p are shown in Table 2. In the C1s, the signal is associated with pi-pi interactions. This is one of the possible ways in which Penicillin G binds to the surface of **MIL-53** and **1** [63].

The O1s peaks coincide with what is reported in the literature [64], and in this case, a shift of the signals is also observed. Fe indicates this ion’s presence in the synthesized MOF and that the Fe(III)-H_2_BDC complex was formed. That is, in the synthesized material, there are unsaturated coordinated Fe sites that can interact with other functional groups related to Fe^3+^.

The same conclusion is reached when the spectrum corresponding to Fe2p was analyzed. The penicillin can be absorbed into the MOF through electrostatic interactions between the Fe ion and the S and N functional groups of Penicillin G. This is confirmed by N1s signals since the characteristic signal of the N-Fe interaction appears at 396 eV [65].

When **MIL-53** is deposited on **BCaP**, the signal characteristics of Ca, P, and other elements coincide with the MOF. For Fe2p, a signal appears at 712.05 eV, which is associated with Fe-PO_4_. In BCaP, hydroxyapatite could have Ca^2+^ vacancies, and these could be occupied by Fe^3+^. The combination was established between the two materials to form the **1** hybrid compound. This statement is confirmed by the signal at 132.09 eV in the P2p spectrum, which was also reported by Carrera et al. [66]. The presence of calcium phosphates from the **BCaP** biomaterial is observed in the Ca2p spectrum when two signals appear at 347.73 eV and 350.93 eV, corresponding to two different environments of **BCaP**; one of these attached to the PO_4_ tetrahedra and the other attached to the -OH of the biomaterial. The channels that are formed by the union of the first Ca with the PO_4_ tetrahedra could serve to confine Penicillin G and are reported to have large lengths [67]. Also, this confirms the interaction between **1** and Penicillin G. Peaks appear that confirm the Fe-S, Fe-N, and Fe-O interactions, which indicate that Penicillin G could bind to **MIL-53** and **BCaP**.

**Table 2:** XPS signals associated with the chemical elements of the synthesized materials are found in the high-resolution spectra.

Elements	Be (eV)	Interactions Associated	Ref.
P2p	132.09	PO_4_-Fe	[66]
C1s	291	π-π	[63]
Ca2p	347.73 350.93	PO_4_^3−^ -OH	[67]
N1s	396	N-Fe	[65]
O1s	532.11 532.76	O-Fe Fe-O-H	[64]
Fe2p	706 712 712.05	Fe-N Fe-S Fe-PO_4_	[68] [69]

**Figure 7:**
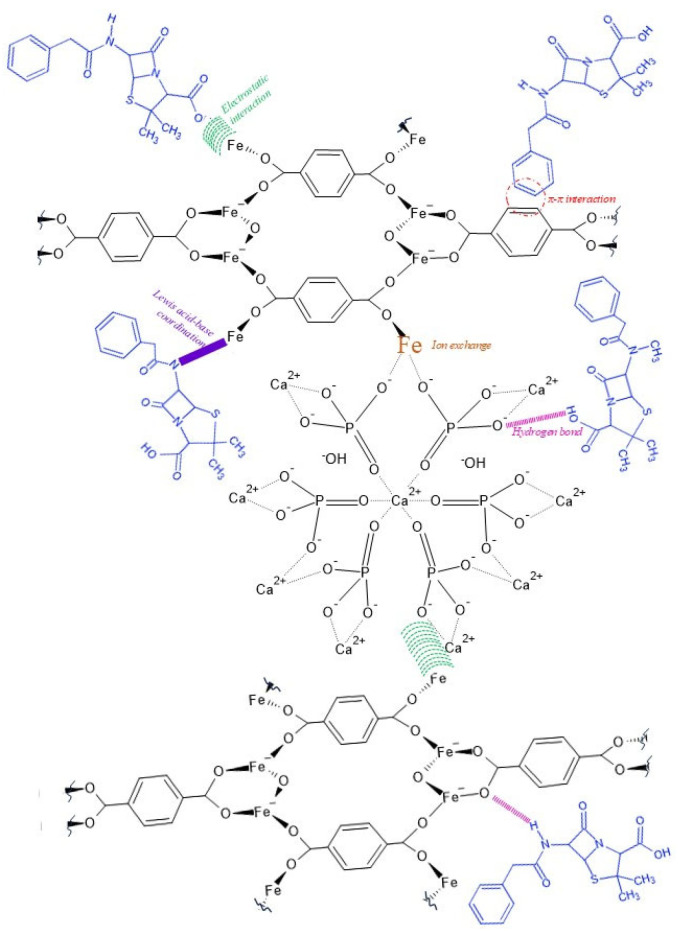
Hypothetical mechanism to the interaction between **MIL-53**, **BCaP**, and Penicillin G molecules.

According to the results obtained by XPS, as well as the confirmation of the modification of the morphology of the materials, a loading mechanism of Penicillin G is proposed, which implies several types of interactions for the drug to be adsorbed on the materials. Figure 7 shows two phases of the loading. The first is related to the MOF **MIL-53** binding to the **BCaP** surface. This happens by occupying the Fe^3+^ of the Ca^2+^ vacancies or electrostatic interactions between the Fe^3+^ (metal center of the MOF), with the most exposed oxygen coming from the phosphate group (PO_4_^3−^) of the **BCaP**. The last stage is binding the drug to the active sites in the material obtained. In this case, as it is a hybrid material, the interactions can be electrostatic pi-pi interactions by the aromatic rings of the MOF and Penicillin G, Lewis acid–base coordination interactions, and hydrogen bridge interactions.

Each XPS showed that iron remains in its oxidation state of interest (3+) and is not affected by either the synthesis or how it binds to the biomaterial or Penicillin G. Therefore, it is important to state that the metal ion in a metal–organic lattice is not altered in its oxidation state and forms typical bonds, just as enterobactin does when it binds to free iron ions in living organisms [70].

All these possible interactions are the ones that have made it possible to load large amounts of Penicillin G into the synthesized materials. Therefore, the potential of these materials for the application in question is confirmed.

**Figure 8:**
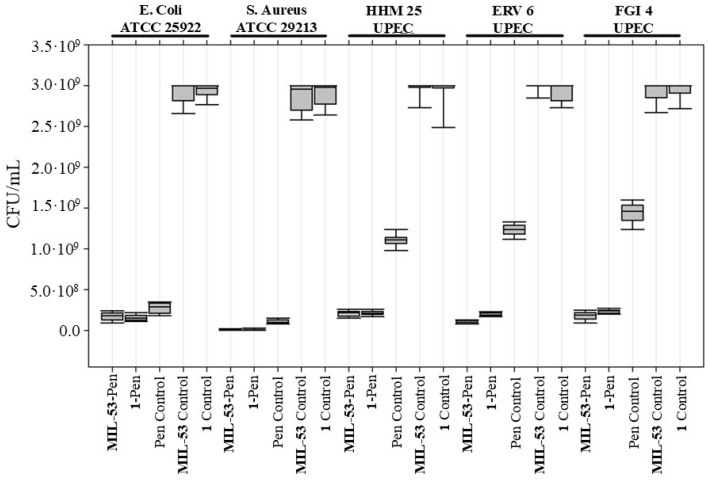
Antibacterial activity test of **MIL-53-Pen** and **1-Pen**. Colony forming units per milliliter (CFU/mL) for *E. coli* strain ATCC 25922, *S. aureus* strain ATCC 29213, and Penicillin-G-resistant UPEC isolates (HHM 25, ERV 6, FGI 4) were compared between treatments with **MIL-53-Pen**, **1-Pen**, and controls: Penicillin G, **MIL-53**, and **1**. Median CFU/mL values are depicted by the central line, with the interquartile range as the box and the range of the data as whiskers.

Some of these interactions facilitate, precisely, the controlled release of Penicillin G, such as hydrogen bridge bonds, and the formation of new functional groups, such as amide bonds [71] (in Figure 8, red lines and purple semicircle, respectively).

### 3.4. Antibacterial Activity Results

The antimicrobial effectiveness of **MIL-53** conjugated with Penicillin G, both with and without the addition of **BCaP**, was evaluated against two bacterial American Type Culture Collection (ATCC) strains (*E. coli* and *S. aureus*) and various Penicillin-G-resistant uropathogenic *E. coli* (UPEC) isolates, HHM 25, ERV 6, and FGI 4. Bacteria were grown in the presence of MOFs in liquid media with agitation before inoculation into agar plates for enumeration of CFUs/mL. This approach maximizes the likelihood of contact between the bacterial cells and MOFs, as mentioned in Wyszogrodzka et al. [72].

Before analyzing the antibiotic-loaded materials, we quantified the intrinsic antibiotic activity of the unloaded frameworks (**MIL-53** and compound **1**). Both produced only marginal CFU reductions across all strains, confirming that the frameworks themselves have negligible antibacterial action under the conditions tested. This baseline establishes that any further reduction in viable counts can be attributed to the antibiotic load.

Treatment groups, therefore, comprised (i) Penicillin G alone, (ii) **MIL-53-Pen** and **1-Pen**, and (iii) the unloaded controls described above. As shown in Figure 8, for the ATCC strains (*E. coli* and *S. aureus*), Penicillin G alone produced a clear antibacterial effect, showing a strong reduction in CFU compared to the unloaded frameworks. However, its performance was very similar to that of **MIL-53-Pen** and **1-Pen**, suggesting that in susceptible strains, MOF loading does not further enhance the effect.

In contrast, the effect was more evident in the Penicillin-G-resistant UPEC isolates (HHM 25, ERV 6, FGI 4). In these strains, Penicillin G alone had little to no effect, while both **MIL-53-Pen** and **1-Pen** notably reduced bacterial counts. This indicates that MOF loading improves antibiotic efficacy specifically against resistant isolates, providing a clear advantage in overcoming resistance in UPEC isolates.

Similar studies utilizing **MIL-53** in conjunction with metals like Ag [73] or antibiotics such as Vancomycin [74] have demonstrated the potent antibacterial capabilities of this type of MOF framework. Our research further underscores the adaptability of **MIL-53** as a platform for antimicrobial applications, particularly highlighting its potential to address antibiotic-resistant bacterial strains. Nonetheless, further investigations are required to fully delineate the specific antibacterial mechanisms underlying our **MIL-53** conjugated with Penicillin G formulations.

**MIL-53-Pen** growth inhibition rates against sensitive strains were 94.0% and 99.7% for *E. coli* ATCC 25922 and *S. aureus* ATCC 29213, respectively (Table 3).

Similar values were reported in the study of Qi et al. [75], where MIL-53 microparticles were used to encapsulate lysozymes, and its antibacterial activity showed inhibition rates of 98.1% against *E. coli* and 99.1% for *S. aureus.* Another study by Lin et al. [74] evaluated the antibacterial activity of Vancomycin (Van) encapsulated in MOF-53 nanoparticles and showed that MOF-53@Van at a concentration of 25 μg/mL (similar to the concentration used in the present study) exhibited a growth inhibition rate of 32.1% against *S. aureus*. In that study, an inhibition rate of 99.3% against *S. aureus* was achieved when the concentration of the MOF was increased to 200 g/mL, representing a 10-fold increase compared to the MOF concentration used in the present study. Ghaffar et al. [76] evaluated the antibacterial activity of MOF MIL-53 loaded with Vancomycin against resistant *S. aureus* strains. The authors demonstrated that the minimum inhibitory concentration (MIC) for this MOF was lower (indicating better antibacterial activity) than that required with Vancomycin alone or the same nanomaterial without the antibiotic. This can be compared to a greater reduction in **MIL-53-Pen** concerning penicillin control in the present study.

## 4. Conclusions

In this study, a metal–organic framework (MOF) from the MIL series was synthesized under mild experimental conditions that do not require sophisticated infrastructure, employing environmentally benign solvents and reagents to ensure biocompatibility and minimize ecological impact. The resulting MOF was subsequently supported on a bioceramic-phosphate (**BCaP**) matrix derived from the sustainable processing of bovine bone. Comprehensive physicochemical characterization confirmed the successful incorporation of the MOF onto the **BCaP** support, as well as the adsorption of Penicillin G. Morphological changes observed after drug loading evidenced the effective incorporation of the antibiotic into the composite materials. X-ray photoelectron spectroscopy (XPS) analyses enabled the identification of the interaction mechanism between Penicillin G and the composite. Although a decrease in crystallinity was observed upon MOF deposition onto the **BCaP** matrix, this structural alteration did not compromise the antibiotic loading capacity or its subsequent release performance.

The **1** material exhibited the highest adsorption capacity for Penicillin G among the evaluated materials and consequently demonstrated the most substantial cumulative drug release over the monitored period. The release kinetics surpassed those previously reported under comparable experimental conditions. The release behavior is attributed to multi-step diffusion-controlled mechanisms, likely influenced by the hierarchical porosity and structural complexity of the hybrid MOF-bioceramic system. The synthesized hybrid materials allow the use of different functional groups, surface defects (vacancies), and metallic centers to load Penicillin G and release it in a controlled way, taking advantage of the new functional groups that are formed.

Antibacterial assays complemented these physicochemical findings. The unloaded **MIL-53** and compound **1** frameworks displayed only marginal intrinsic activity, confirming that the carriers themselves are effectively inert under the test conditions. When loaded with Penicillin G, however, both **MIL-53-Pen** and **1-Pen** matched the performance of free Penicillin G against drug-susceptible ATCC strains, indicating that the carriers do not impede drug action. Importantly, against Penicillin-G-resistant uropathogenic *E. coli* isolates, the loaded MOFs achieved additional log-scale reductions in viable counts that free Penicillin G alone could not attain. These outcomes underscore a synergistic interplay between the MOF matrix and the antibiotic cargo, offering a potential route to extend the therapeutic reach of β-lactams.

## Figures and Tables

**Table 1:** Kinetic model parameters and degree of burst for **MIL-53** and **1**.

Materials	MIL-53	1
Zero-order model		
k_0_ (%·days^−1^)	0.2976	0.2400
R^2^	0.9811	0.9766
First-order model		
k_f_ (days^−1^)	0.6724	0.4906
R^2^	0.9908	0.9797
Higuchi model		
k_H_ (%·days^−0.5^)	0.7313	0.5943
R^2^	0.9778	0.9794
Degree of burst		
(ΔMt/Δt) to t = 1 day	23.0154	8.7652
(ΔMt/Δt)_SS_ to equilibrium time	81.0666 (t = 6 days)	31.1073 (t = 4 days)
DB (%)	28.39	28.18

**Table 3:** Growth inhibition rates are expressed as the percentage of reduction in CFUs/mL after MOF treatment. Percentages were calculated with the average values of CFUs/mL of each treatment divided by the average values of CFUs/mL of controls. Standard deviations are also depicted in the table.

Treatment vs. Control	Percentage of Reduction of CFUs/mL (%)				
	*E. coli* ATCC 25922	*S. aureus* ATCC 29213	HHM25 UPEC	ERV6 UPEC	FGI4 UPEC
MIL-53-Pen versus Penicillin G Control	37 ± 14.3	93 ± 6.4	81 ± 2.6	92 ± 1.3	88 ± 2.8
1-Pen versus Penicillin G control	43 ± 2.5	93 ± 6.4	81 ± 1.9	84 ± 1.3	84 ± 1.4
MIL-53-Pen versus Growth Control	94 ± 1.3	100 ± 0.2	93 ± 1.0	96 ± 0.5	94 ± 1.3
1-Pen versus Growth control	95 ± 0.2	100 ± 0.2	93 ± 0.7	93 ± 0.5	92 ± 0.7

## Data Availability

Data will be made available on request.
